# Research on the Design and Control Method of Robotic Flexible Magneto-Rheological Actuator

**DOI:** 10.3390/s25226921

**Published:** 2025-11-12

**Authors:** Ran Shi, Sheng Jian, Guangzeng Chen, Pengpeng Yao

**Affiliations:** 1 School of Undergraduate Education, Shenzhen Polytechnic University, Shenzhen 518055, China; shiran@szpu.edu.cn; 2 School of Mechanical and Electrical Engineering, Shenzhen Polytechnic University, Shenzhen 518055, China; jiansheng@szpu.edu.cn; 3 School of Intelligence Science and Engineering, Harbin Institute of Technology, Shenzhen 518055, China; 13838134724@163.com

**Keywords:** flexible robotic joint, magneto-rheological fluid clutch, joint torque control

## Abstract

To meet the safety and compliance requirements pertaining to robots when interacting physically with humans or the environment in unstructured settings such as households and factories, in this study, we focus on methods for the design and control of a flexible robotic magneto-rheological actuator (MRA). Firstly, for the magneto-rheological fluid clutch (MRC), which is the core component of the MRA, an equivalent magnetic circuit model was established to accurately calculate the magnetic field inside the clutch, and a thermal circuit model was constructed to analytically determine the operating temperature of each component. Considering practical engineering constraints, including mechanical structure, magnetic saturation, maximum current, and maximum temperature, a genetic algorithm was used to optimize parameters of the MRC. Secondly, based on the dynamic characteristics of the MRA, a dynamic model incorporating the motor, reducer, MRC, and load link was established. Given scenarios where torque sensors cannot be installed due to cost and structural space limitations, a model reference PID feedforward control strategy was designed. Torque was estimated using input current. Finally, an experimental platform was built, and static and dynamic torque output experiments were conducted. These experiments verified the excellent torque tracking performance of the designed MRA. Through multi-physics modeling, parameter optimization, and control strategy design, this paper provides a solution for flexible robotic joints that integrates high torque, high compliance, and safety.

## 1. Introduction

The development of robots is advancing at a rapid pace, and demand is expanding from industry to services and special applications. For instance, quadruped robots are used to explore rough terrain and transport supplies, exoskeleton robots are worn on the body to assist with walking, rescue robots enter disaster-stricken areas for emergency rescue and disaster relief, and humanoid robots perform tasks such as serving tea and pouring water [1]. In these applications, robots move away from structured and known environments and enter unstructured environments such as unknown mountainous areas, forests, cities, workshops, and households. When robots need to interact physically with humans or the environment, they must ensure the safety of humans, themselves, and objects. Meanwhile, robots also need to avoid self-damage under the impact force generated during physical interactions with humans, the environment, or objects.

Robots’ joints are their most important executive mechanisms, and their characteristics directly determine a robot’s performance, safety, and application scenarios. Traditional robot joints consist of a motor connected in series with a reducer with a large reduction ratio. Through high-stiffness mechanism design and position control algorithms, they provide large driving torque for industrial robots and ensure motion accuracy in production and processing. However, this design significantly reduces robots’ compliance and safety, greatly limiting their applications in unstructured environments such as workshops and households [2].

**Figure 1:**
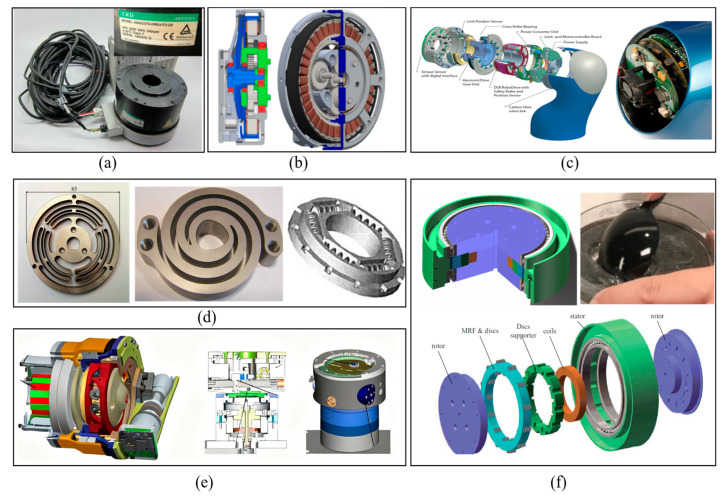
Main solutions for compliant robot actuators. (**a**) Direct-drive actuator (DDA); (**b**) quasi-direct-drive actuator (QDA); (**c**) integrated joint; (**d**) series elastic actuator (SEA); (**e**) variable-stiffness actuator (VSA); (**f**) magneto-rheological actuator (MRA).

The literature presents several prominent actuator architectures designed to enhance the compliance of robotic joints, as summarized in Figure 1. Direct-drive actuators (DDA) and quasi-direct-drive actuators (QDA) forgo or minimize reduction gears to achieve high backdrivability, but this often comes at the cost of limited output torque, restricting their use in high-load scenarios [3,4]. Robotic integrated joints compactly combine motors, reducers, and drivers, relying on sophisticated sensor feedback for compliance [5]. A common strategy for introducing passive compliance is the use of series elastic actuators (SEA), which integrate a fixed elastic element in series with the output [6,7]. Evolving from SEA, variable-stiffness actuators (VSA) incorporate an additional mechanism to actively modulate the joint stiffness online, though this often leads to increased mechanical complexity [8,9].

In Figure 1f, magneto-rheological actuators (MRAs) incorporate a magneto-rheological fluid clutch (MRC) in series at the end of traditional robot joints [10,11,12]. The stiffness of an MRA is adjusted online by changing the engagement force between the stator and rotor of the MRC. MRAs do not require elastic elements and feature advantages such as simple structure, fast response, excellent compliance, and impact resistance. These characteristics have attracted widespread attention among researchers. Mousavi [13] designed a T-shaped drum-type MRC and successfully applied it to prosthetic knee joints. In their study, a zoned modeling approach was conducted for the MRC, and the relationship between the MRC’s output torque and its structural parameters was derived. Viau [14] applied an MRC to a cable-driven robotic arm with two degrees of freedom, endowing the robot with excellent compliance. To ensure safe physical interaction between robots and humans, Ahmed [15] designed a simple two-degrees-of-freedom robotic arm using two MRCs with a maximum torque of approximately 10 Nm. Fauteux [16] proposed a dual-differential MRC, which uses a motor–reducer system to drive two MRCs in opposite directions; the engagement forces of the two clutches are coupled and then transmitted to the connecting rod. The dual-differential MRC only requires the motor–reducer system to maintain unidirectional motion at all times and realizes torque output in both positive and negative directions by controlling the current of the two MRCs, thereby eliminating gear backlash. Gudmundsson [17,18,19] used finite element analysis for the multi-objective optimization design of MRC and successfully applied the optimized clutches to prosthetic knee joints. Shafer, Kermani, and Yadmellat conducted a comparative simulation study on the compliance and safety of MRAs, KUKA LWR III robots, SEAs, and DDAs. The results verified that MRAs exhibit better compliance and safety than SEAs in human–robot collaboration [20]. They also studied modeling and controlling the hysteresis between the current and torque of MRCs using the Preisach model, artificial neural networks, and embedded magnetic sensors [21,22] and proposed an active distributed semi-drive transmission method. This method only requires one motor–reducer system to achieve independent control of multiple degrees of freedom through oppositely distributed MRCs, thereby significantly reducing robot costs. Li [23] introduced an untethered MRF robot that employed an innovative approach by encapsulating the MRF within an elastic membrane. St-Jean [24] showed that the safety level of collaborative robots can be increased by a factor up to 3 by changing the conventional servo-geared actuator architectures for MR actuators alone. Finally, Li [25] presented a flexible safety system that used MRF-based joints for the purposes of limb rehabilitation. The joints could control the upper limb rehabilitation system to achieve the desired effect by increasing and changing the patient’s interactive force.

The technical challenges in designing and controlling MRAs lie in the design and control of MRCs. Although MRAs have attracted the attention of scholars worldwide, relevant research remains relatively limited compared to other types of robot joints. The integrated modeling, optimization, and control methods applicable to MRAs still require investigation. This paper focuses on the design and control of a flexible robotic MRA. The innovation of this study lies in conducting multi-physics modeling on the MRC, the core component of the MRA, by combining an equivalent magnetic circuit model and a thermal circuit model. The MRC’s parameters are optimized through a genetic algorithm to meet engineering constraints, and a model reference-based PID feedforward control strategy is designed. Finally, experiments verify that the designed MRA has excellent torque tracking performance, providing an integrated solution with high torque, high compliance, and safety for flexible robotic joints.

The subsequent structure of this article is as follows: Section 2 primarily addresses the structural design and parameter optimization of the MRA. Section 3 describes dynamic modeling analysis and torque control research. In Section 4, through static and dynamic torque output experiments, it is verified that the designed MRA exhibits excellent torque tracking performance.

## 2. MRA Design and Optimization

In this section, the magnetic field inside the clutch is accurately calculated using an equivalent magnetic circuit model. Then, a thermal circuit model of the clutch is established, and the operating temperature of each component of the clutch is calculated analytically. Finally, based on the magnetic circuit model and thermal circuit model, and considering actual engineering constraints, the structural design and parameter optimization of the MRA are completed, resulting in a safety-oriented design with optimal torque performance.

### 2.1. Structural Design of MRA

**Figure 2:**
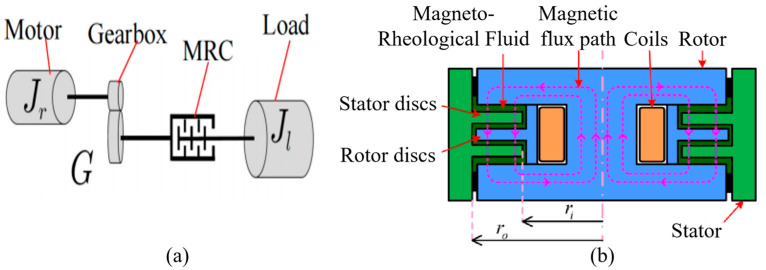
Basic structure of MRA. (**a**) MRA structure; (**b**) MRC structure.

As shown in Figure 2a, the MRA consists of a traditional robot joint motor and a reducer, with an MRC connected in series. The stator of the MRC is fixedly connected to the output shaft of the reducer and rotates with it, while the rotor of the clutch is connected to the robot load. The working principle of the MRC is illustrated in Figure 2b. Its main components include a stator, stator disks, a rotor, rotor disks, coils, and magneto-rheological fluid filling the gaps between the stator and rotor disks. The stator disks are fixedly attached to the stator, and the rotor disks are fixedly attached to the rotor. By adjusting the magnitude of the current in the excitation coil, the intensity of the magnetic field acting on the MRC can be regulated, which in turn changes the solidification degree of the MRC. This generates varying engagement forces between the MRC’s stator and rotor disks. Thus, the MRC can transmit different torques to the link according to the current level in the excitation coil. Due to the MRC’s fast response and its ability to undergo continuous and reversible transitions between a liquid state and various semi-solid states, the MRA can achieve rapid and continuous control of torque ranging from very small to very large values while exhibiting excellent compliance and impact resistance.

### 2.2. Magnetic Circuit Model Analysis of MRC

As shown in Figure 2b, given the inner radius $r_{i}$ and outer radius $r_{o}$ of the effective working area of the MRC, the engagement torque $T_{c}$ of the MRC can be calculated using the Bingham model [26,27] as follows:

(1) $$T_{c}=2N\pi \int_{r_{i}}^{r_{o}}\tau (B(x))x^{2}dx,$$

where τ(·) is the shear stress of magneto-rheological fluid under different magnetic field strengths; B(x) is the nonlinear magnetic induction intensity in magneto-rheological fluid, which is distributed nonlinearly along the radial direction; and N is the number of magneto-rheological fluid gaps. Assuming the number of rotor disks is $N_{r}$, and the number of stator disks is $N_{s}$, then $N=2N=2(N_{r}+1)$. $r_{i}$ is the inner diameter of the annular stator disks, and $r_{o}$ is the outer diameter of the annular rotor disks.

The shear stress of the magneto-rheological fluid can be calculated as

(2) $$\tau (B(x))=\tau_{0}(B(x))+\eta (B(x))\dot{\gamma }(x),$$

where $\tau_{0}(\cdot )$ is the yield stress of the magneto-rheological fluid under different magnetic field strengths and η(·) is the viscosity coefficient of the magneto-rheological fluid under different magnetic field strengths. $\dot{\gamma }(x)$ is the shear rate of the magneto-rheological fluid at radius x and can be expressed as

(3) $$\dot{\gamma }(x)=\frac{({\dot{\theta }}_{m}G-{\dot{\theta }}_{L})x}{h},$$

where h is the thickness of the magneto-rheological fluid gap; ${\dot{\theta }}_{m}$ is the motor speed of the magneto-rheological fluid joint; ${\dot{\theta }}_{L}$ is the rotational speed of the load-connecting rod; and G is the reduction ratio of the reducer.

When the average magnetic induction intensity $\bar{B}$ in the magneto-rheological fluid is selected to replace the nonlinearly distributed magnetic induction intensity B(x) in Equation (1) for approximate calculation, the clutch torque $T_{c}$ of the magneto-rheological fluid clutch can be approximated as

(4) $$\begin{array}{cc} T_{C} & =2N\pi \int_{r_{i}}^{r_{o}}r(B(x))x^{2}dx\\ & =2N\pi \int_{r_{i}}^{r_{o}}r_{0}(B(x))x^{2}dx+2N\pi \int_{r_{i}}^{r_{o}}\eta (B(x))\dot{\gamma }(x)x^{2}dx\\ & \approx \frac{2}{3}\pi N\tau (\bar{B})(r_{o}^{3}-r_{i}^{3})+\frac{\pi \eta (\bar{B})}{2h}N\left(\frac{{\dot{\theta }}_{m}}{G}-{\dot{\theta }}_{L}\right)(r_{o}^{4}-r_{i}^{4})\\ & ≜T_{B}+T_{\eta } \end{array},$$

where $T_{B}$ is the magneto-rheological torque and $T_{\eta }$ is the viscous torque. Therefore, the output torque of the magneto-rheological fluid joint acting on the load-connecting rod can be expressed as

(5) $$T_{L}=\mathrm{sign}\left(\frac{{\dot{\theta }}_{m}}{G}-{\dot{\theta }}_{L}\right)T_{C},$$

where sign(·) is the sign function. The symbols and descriptions of the magnetic circuit model are shown in Table 1.

### 2.3. Thermal Circuit Model Analysis of MRC

When an MRC is used as the power source in a magneto-rheological fluid joint, the relative speed between the rotating disk and the fixed disk of the magneto-rheological fluid clutch can be very low, or even zero. Therefore, the frictional heat generated by the relative friction between the rotor and the stator is extremely small and can be neglected. The heat generation of the magneto-rheological fluid clutch in the magneto-rheological fluid joint is mainly caused by the Joule heat produced when an electric current is applied to the excitation coil. If the magneto-rheological fluid clutch is unreasonably designed and the heat generated by the coil cannot be dissipated in a timely manner, it will lead to an excessively high temperature of the coil, which may result in burnout. Additionally, the magneto-rheological fluid will undergo changes in its properties due to the excessively high temperature, causing the clutch to fail to work normally. Therefore, it is essential to establish a thermal circuit model for the magneto-rheological fluid clutch and take its heat generation into account during the design process so as to ensure that the magneto-rheological fluid clutch can operate safely and reliably for an extended period.

The thermal circuit model is established by dividing the magneto-rheological fluid clutch into regions based on the shape and material properties of the components within the clutch. When constructing the thermal circuit model of the magneto-rheological fluid clutch based on Ohm’s Law for Thermal Circuits, heat is generated in the excitation coil and transferred to the surrounding air through two paths: one is the axial direction, where heat is dissipated to the air via the rotor at the upper and lower ends to the surface of the magnetic yoke; the other is the radial direction, where heat is dissipated to the air from the circumferential surface of the stator.

Let the total amount of heat dissipated along the axial direction be $P_{cap}$ and the total amount of heat dissipated along the radial direction be $P_{m}$. The total heat generated by the excitation coil is $I^{2}R$. Then, according to the law of conservation of energy, the following equation can be obtained:

(6) $$\left\{\begin{array}{l} I^{2}R=P_{m}+P_{cap}\\ P_{m}=\frac{t_{0}-t_{m}}{\Omega_{ALgap}+\Omega_{AL}+\Omega_{m//}}+\frac{t_{cap}-t_{m}}{\Omega_{m⊥}}\\ P_{cap}=\frac{t_{0}-t_{cap}}{\Omega_{cgap}+\Omega_{c}+\Omega_{cap}}-\frac{t_{cap}-t_{m}}{\Omega_{m⊥}} \end{array}\right.,$$

where $\Omega_{*}$ represents the thermal resistance of each part, $t_{0}$ is the temperature of the excitation coil, $t_{m}$ is the temperature of the magneto-rheological fluid, and $t_{cap}$ is the surface temperature of the rotor.

There are two ways for the magneto-rheological fluid clutch to dissipate heat to the air: natural convection and radiative heat dissipation. Based on Ohm’s Law for Thermal Circuits, the following closed thermal circuit equation can be derived [28]:

(7) $$\begin{array}{ll} I^{2}R= & \left[hA_{t_{m}}\left(t_{m}-t_{\infty }\right)+\varepsilon \sigma A_{t_{m}}\left(t_{m}^{4}-t_{\infty }^{4}\right)\right]\\ & +\left[hA_{t_{cap}}\left(t_{cap}-t_{\infty }\right)+\varepsilon \sigma A_{t_{cap}}\left(t_{cap}^{4}-t_{\infty }^{4}\right)\right] \end{array},$$

where h is the convection coefficient, $A_{t_{m}}$ is the mantle surface area of the stator for radial heat dissipation (m^2^), $A_{t_{cap}}$ is the total end-cap surface area of the rotor/yoke for axial heat dissipation (m^2^), σ is the Boltzmann constant, ε is the radiative emissivity coefficient, and $t_{\infty }$ is the ambient air temperature.

Given the limit temperature $t_{0}$ of the coil and the ambient temperature $t_{\infty }$, the maximum current $I_{max}$ for the magneto-rheological fluid clutch to operate safely and reliably, the maximum temperature $t_{m}$ of the magneto-rheological fluid, and the temperature of the rotor yoke $t_{cap}$ can be solved by simultaneously solving Equations (6) and (7). Since the heat conduction areas $A_{t_{m}}$ and $A_{t_{cap}}$, the thermal resistances of each part, and the coil resistance R are all calculated based on the structural parameters of the magneto-rheological fluid clutch, the calculated maximum current $I_{max}$, maximum temperature $t_{m}$ of the magneto-rheological fluid, and temperature $t_{cap}$ of the rotor yoke are inevitably closely related to the structural parameters of the magneto-rheological fluid clutch. By using the calculated results to constrain the structural parameters of the magneto-rheological fluid clutch in the optimal design process, a safe and reliable design of the magneto-rheological fluid clutch can be obtained.

The magnetic circuit model and the thermal circuit model are not independent; they form a coupled multi-physics system. Understanding their coupling relationship is essential for accurate modeling and reliable design. The excitation current is the common input to both models. In the magnetic circuit, the excitation current generates the magnetomotive force, which determines the magnetic flux density in the MR fluid and the core, ultimately defining the output torque via Equation (1). Simultaneously, according to the thermal circuit model (Equations (6) and (7)), the same current flowing through the coil resistance produces Joule heating, which is the primary heat source in the system. This heat leads to a rise in the temperatures of the coil, the MR fluid, and other components. The constraints on maximum current and maximum temperature are interdependent. The genetic algorithm finds a set of structural parameters that simultaneously satisfy the torque requirement from the magnetic model without exceeding saturation flux density, while ensuring that the resulting Joule heating, calculated by the thermal model, does not cause temperatures to exceed safe limits for the coil and the MR fluid. This integrated approach ensures that the optimized MRC delivers the required performance reliably under continuous operation.

### 2.4. Parameter Optimization Design of MRA

The goal of optimizing the design of the magneto-rheological fluid clutch is to select appropriate values for the inner radius $r_{i}$ of the annular magneto-rheological fluid, the radius $r_{c}$ of the rotor core, the number of turns n of the excitation coil, and the input current I under the constraints of the clutch’s mechanical structure, magnetic saturation, maximum current, and maximum temperature. This selection aims to enable the magneto-rheological fluid clutch to achieve the maximum torque output. The design variables and their parameter design ranges are expressed as follows:

$$r_{i}\in [13,42.5]\;\mathrm{mm},\;\;r_{c}\in [4,33.5]\;\mathrm{mm},\;\;n\in [15,1000],\;I\in [0.5,3.0]\;A.$$

The optimization design problem of the magneto-rheological fluid clutch described above can be mathematically formulated as

(8) $$\left\{\begin{array}{cc} \underset{r_{i},r_{c},n,I}{\max}T & =2N\pi \int_{r_{i}}^{r_{o}}r(B)r^{2}dr\\ s.t. & B-1.2\leq 0\\ & \Phi_{c}-2.2\times \pi (r_{c}^{2}-r_{ho}^{2})\leq 0\\ & I-I_{max}\leq 0\\ & t_{m}-130\leq 0\\ & r_{ho}-r_{c}<0\\ & r_{c}+l_{a}-r_{i}=0\\ & h-(h_{c}+2h_{y})=0 \end{array}.\right.$$

The magnetic induction intensity in the magneto-rheological fluid is calculated by the magnetic circuit model (4), while the maximum current $I_{max}$ and the operating temperature $t_{m}$ of the magneto-rheological fluid are calculated by the thermal circuit model (7) proposed in this chapter. The genetic algorithm is adopted to solve this optimization problem, and the obtained global optimal solution is as follows:

$$\left[r_{i},r_{c},n,I\right]=\left[35.7\;\mathrm{mm},19.5\;\mathrm{mm},268,2.0\;A\right].$$

All the results obtained fall within the range of the design variables; therefore, the results can be considered to have converged to the global optimal solution. The thickness of the rotor yoke is $t_{y}$ = 10.5 mm ≈ 11 mm. It should be noted that these solved optimal parameters have all been rounded to meet the practical requirements of mechanical processing.

The rounded solution obtained is substituted into the equivalent magnetic circuit model, the thermal circuit model, and the Bingham model to calculate the maximum magnetic induction intensity $B_{m}$ in the magneto-rheological fluid, the maximum magnetic induction intensity $B_{c}$ of the iron core, the maximum magnetic induction intensity $B_{rc}$ in the rotor-to-yoke at radius $r_{c}$, and the maximum torque T of the designed magneto-rheological fluid clutch. The results are shown in Table 2.

**Figure 3:**
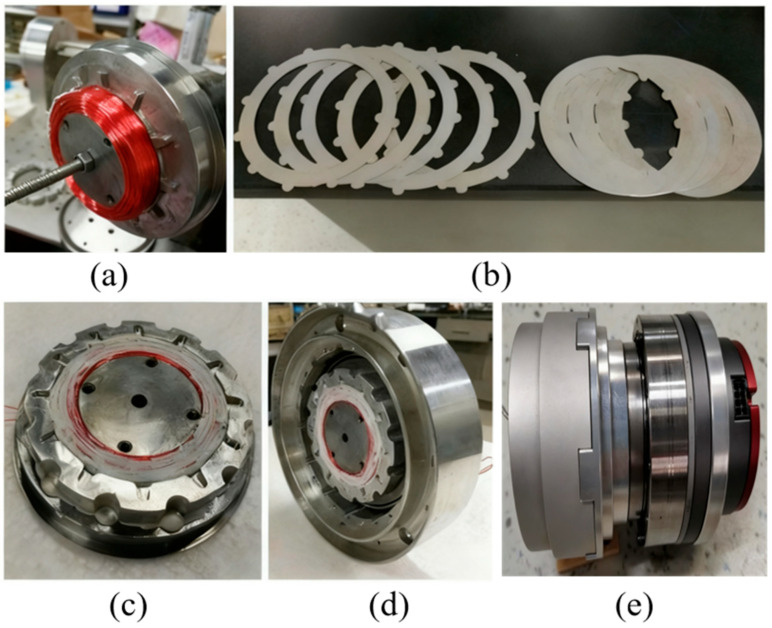
Prototype of the MRA. (**a**) Winding coils on the rotor core; (**b**) stator disks and rotor disks; (**c**) assembling the disk supporter on the rotor; (**d**) assembling the rotor into the stator; (**e**) prototype of the MRA (including the motor, reducer, and MRC).

The specific structure of the built-in coil disk-type magneto-rheological fluid clutch designed in this paper is shown in Figure 3. It consists of a pair of pure iron rotors, an aluminum alloy stator, a set of copper coils, an aluminum alloy disk skeleton, a number of annular rotating disks and stationary disks made of silicon steel sheets, and magneto-rheological fluid filling the gaps between the rotating and stationary disks.

When current is applied to the coil, the coil generates closed magnetic field lines inside the magneto-rheological fluid clutch. These magnetic field lines pass through the iron core of the rotor pair, the yoke of the rotor pair, the magneto-rheological fluid, and the rotating/stationary disks before finally returning to the iron core of the rotor pair to form a closed loop. When an external magnetic field acts on the magneto-rheological fluid, the fluid can transform from a liquid state to a solid-like state in an extremely short time (usually a few milliseconds). The degree of solidification is positively correlated with the strength of the external magnetic field, which creates resistance to the relative motion between the rotating and stationary disks immersed in it. Thus, a current-controllable engagement torque is formed between the rotor and stator of the magneto-rheological fluid clutch. A through-hole is machined in the middle of the rotor pair to facilitate the hollow wiring of the magneto-rheological fluid joint.

## 3. Dynamic Modeling and Torque Control of the MRA

### 3.1. Dynamic Modeling of the MRA

**Figure 4:**
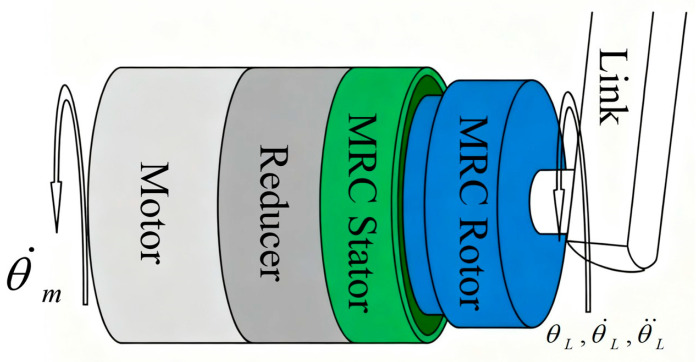
Structures of the MRA.

A dynamic model of the robot link, driven by external forces and the magneto-rheological fluid joint, is shown in Figure 4. Assume that the motor in the magneto-rheological fluid joint rotates at a speed of ${\dot{\theta }}_{m}$ and transmits the driving force to the stator end of the magneto-rheological fluid clutch through a reducer with a reduction ratio of G. The rotor end of the magneto-rheological fluid clutch is connected to the link with an inertia of I. The inertia of the magneto-rheological fluid clutch rotor is much smaller than that of the link and can be ignored. When the external force torque is $T_{ext}$ and the clutch torque is $T_{MRC}$, the position, velocity, and acceleration of the link are $\theta_{L}$, ${\dot{\theta }}_{L}$, and ${\ddot{\theta }}_{L}$, respectively. The dynamic equation of the MRA is

(9) $$\left\{\begin{array}{l} I{\ddot{\theta }}_{L}+c(\theta_{L},{\dot{\theta }}_{L}){\dot{\theta }}_{L}+n(\theta_{L},{\dot{\theta }}_{L})=T_{\mathrm{MRA}}+T_{ext}\\ T_{\mathrm{MRA}}=\mathrm{sign}({\dot{\theta }}_{G}-{\dot{\theta }}_{L})|T_{\mathrm{MRC}}| \end{array}\right.,$$

where $c(\theta_{L},{\dot{\theta }}_{L})$ is the link damping coefficient, $n(\theta_{L},{\dot{\theta }}_{L})$ is the link gravity term, ${\dot{\theta }}_{G}$ is the output speed of the reducer, and $T_{\mathrm{MRA}}$ is the output torque of the MRA.

The control inputs of the system include the rotational speed of the motor–reducer and the clutch torque of the magneto-rheological fluid clutch, while the outputs of the system are joint torque, link speed, or link angle. Therefore, this system is a two-input, single-output dynamic system. The clutch torque of the magneto-rheological fluid clutch and the motor–reducer rotational speed shall satisfy

(10) $$\left\{\begin{array}{l} T_{\mathrm{MRC}}=|T_{\mathrm{MRA}}|\\ \mathrm{sign}({\dot{\theta }}_{G}-{\dot{\theta }}_{L})=\mathrm{sign}(T_{\mathrm{MRA}}) \end{array}\right.,$$

where ${\dot{\theta }}_{G}-{\dot{\theta }}_{L}$ has the same sign as $T_{\mathrm{MRA}}$. To solve for this, we can let

(11) $${\dot{\theta }}_{G}-{\dot{\theta }}_{L}=\kappa T_{\mathrm{MRA}},$$

where κ>0.

(12) $$\left\{\begin{array}{l} {\dot{\theta }}_{G}={\dot{\theta }}_{L}+\kappa T_{\mathrm{MRA}}\\ {\dot{\theta }}_{G}-{\dot{\theta }}_{L}=\kappa sign(T_{MRA})\\ {\dot{\theta }}_{G}={\dot{\theta }}_{L}+\kappa sign(T_{MRA}) \end{array}\right.,$$

From (12), let ${\dot{\theta }}_{G}-{\dot{\theta }}_{L}=K(T_{MRA})$, where K(·) is a non-decreasing function passing through the zero point, satisfying $\frac{dK(T_{MRA})}{dT_{MRA}}\geq 0$ and K(0)=0.

### 3.2. Torque Control of the MRA

**Figure 5:**
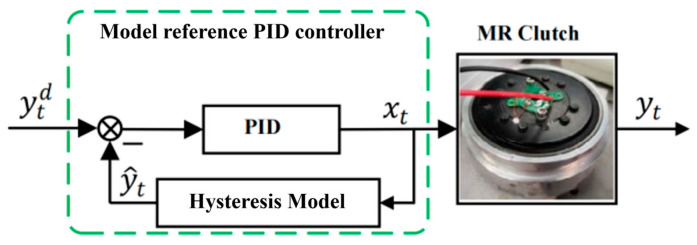
Model reference PID controller.

In magneto-rheological fluid joints, due to constraints on cost and structural space, torque sensors cannot be equipped to measure the clutch torque of the magneto-rheological fluid clutch in many application scenarios. Therefore, the PID control method based on torque feedback is not applicable here. In this paper, Equation (5) is used to model the torque of the magneto-rheological fluid clutch, so that the input current data of the magneto-rheological fluid clutch can be utilized to accurately estimate its torque. By treating this model as the “real magneto-rheological fluid clutch” and using the model’s calculated output as feedback, PID control of the model can be achieved. Meanwhile, the output of the PID controller can be used as the input current of the actual magneto-rheological fluid clutch, thereby achieving its open-loop torque control. A block diagram of such model-based PID feedforward control is shown in Figure 5. Model reference PID feedforward control is a type of open-loop control. Its control accuracy depends on two aspects: the parameters of the PID controller on the one hand and the modeling accuracy of the target object’s model on the other.

Assume that the Laplace transform of the hysteresis model used in Figure 5 is H(s), where X(s) is the Laplace transform of the input current x(t). The error between the output of the hysteresis model and the desired input $Y^{d}(s)$ can be expressed as $E(s)=Y^{d}(s)-\hat{Y}(s)$. It is known that the Laplace transform of the PID controller is $C(s)=K_{p}+K_{I}/s+K_{D}S$. Then, according to the control block diagram in Figure 5, we can obtain

(13) $$\frac{\hat{Y}(s)}{Y^{d}(s)}=\frac{C(s)H(s)}{1+C(s)H(s)}.$$

The control error is

(14) $$E(s)=\frac{Y^{d}(s)}{1+C(s)H(s)}=\frac{sY^{d}(s)}{s+(K_{D}s^{2}+K_{P}s+K_{I})H(s)}.$$

Assuming that the system is stable, according to the Final Value Theorem of Laplace transform, the steady-state error of the system can be obtained as

(15) $$\underset{t\to +\infty }{\lim}e_{\mathrm{ss}}(t)=\underset{s\to 0}{\lim}sE(s)=\underset{s\to 0}{\lim}\frac{s^{2}Y^{d}(s)}{s+(K_{D}s^{2}+K_{P}s+K_{I})H(s)}.$$

When a step-desired output $Y^{d}(s)=1/s$ is given, at this time,

(16) $$\underset{s\to 0}{\lim}sE(s)=\underset{s\to 0}{\lim}\frac{s}{s+(K_{D}s^{2}+K_{P}s+K_{I})H(s)}=0.$$

## 4. Experimental Verification of the MRA

### 4.1. Introduction to the Experimental Platform

**Figure 6:**
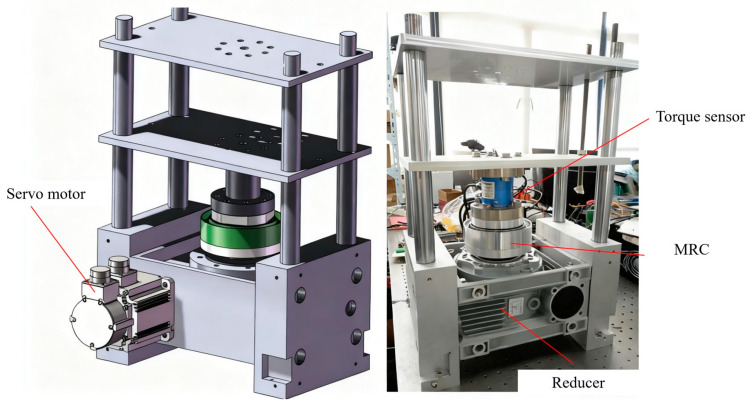
MRC experimental platform.

The MRC experimental platform is shown in Figure 6. In the experimental platform, the stator of the magneto-rheological fluid clutch is connected to the output shaft of a worm gear reducer with a reduction ratio of 100:1, and the rotor is connected to one end of a torque sensor. The input shaft of the worm gear reducer is connected to a 1 kW Panasonic servo motor. The other end of the torque sensor is fixed to the base of the test platform, with a torque measurement range of [0, 500] Nm. The Panasonic servo motor is controlled by its supporting driver via analog signals, and the coil current of the magneto-rheological fluid clutch is controlled by a DC controller set in current control mode. All control signals are sent by the real-time dSPACE 1103 controller. The measured values of the torque sensor and the coil current values of the magneto-rheological fluid clutch are measured, filtered, and then collected and recorded in real time by the real-time dSPACE controller from dSPACE GmbH.

### 4.2. Static Torque Output Experiment

To evaluate the steady-state torque control accuracy and long-term stability of the designed MRA, a long-duration step torque response experiment was conducted in this subsection. A model reference PID controller, based on the fractional-order multi-state differential model, was implemented in the dSpace 1103 control system. The controller cycle was set to 1 ms, with the proportional, integral, and derivative gains of the PID controller set to 0.005, 0.5, and 0, respectively. The system demonstrated remarkable stability over a prolonged 100 s operation, with the steady-state tracking error tightly bounded within ±0.5 Nm (<0.33% of the full-scale range). The Root Mean Square Error (RMSE) was calculated to be 0.21 Nm.

**Figure 7:**
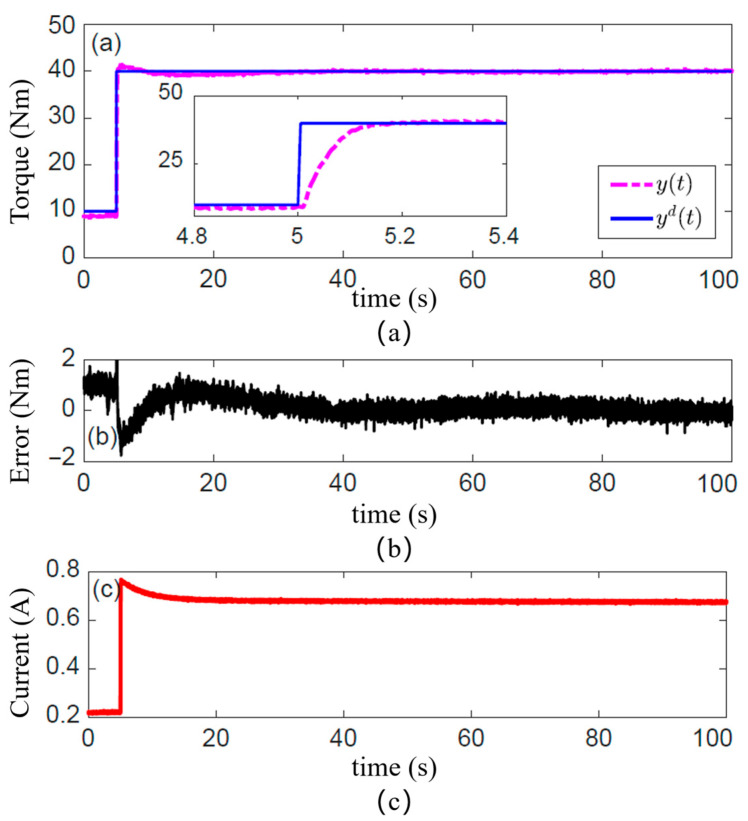
Control performance with long-term step-desired torque. (**a**) Tracking performance of the clutch torque; (**b**) Tracking torque error of the clutch; (**c**) Calculated input current of the model reference PID controller.

The clutch torque obtained under the model reference PID feedforward control, in response to a sustained step-desired torque, is presented in Figure 7. Figure 7a demonstrates the tracking performance of the clutch torque. The solid blue line represents the desired torque. The pink/magenta dashed line represents the measured clutch torque. The response is rapid and settles without clearly overshooting, indicating that the established magnetic circuit model and the Bingham model accurately capture the system’s steady-state gain. More importantly, over the extended period of 100 s, the output torque maintains precise tracking of the desired value without any observable drift or decay.

The long-term stability is further corroborated by the torque tracking error plotted in Figure 7b. The steady-state error remains tightly bound within ±0.5 Nm throughout the entire test duration. And a standard deviation of 0.18 Nm, with the vast majority of errors confined within a range of ±0.4 Nm. This result provides strong validation for the effectiveness of our proposed thermal circuit model and the optimization design. It confirms that the Joule heating from the continuously energized coil is effectively managed, with the internal temperature of the actuator controlled within a safe range. This prevents significant torque drift that would otherwise occur due to the reduction in the MR fluid’s yield stress with rising temperature. Furthermore, it demonstrates that the model-reference control strategy compensates for the inherent, time-dependent torque creep nonlinearity of the MR fluid.

Figure 7c shows the input current calculated by the model reference PID controller. At the instant the step-desired torque is commanded, the current surges to a high value to rapidly build the magnetic field and drive the torque to its target. Subsequently, the current exhibits a creep-like decay before settling to a lower steady-state value. This behavior offers a clear window into the controller’s compensation mechanism. The initial high current is necessary to overcome static friction and achieve fast torque buildup. The subsequent gradual decrease represents an active compensation, guided by the model, for the “torque creep” effect caused by magnetic flux relaxation or microstructural changes in the MR fluid, thereby maintaining high-precision steady-state torque output.

**Figure 8:**
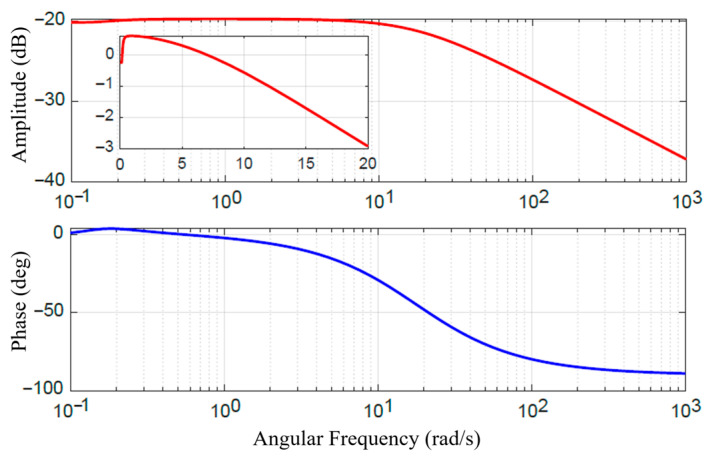
Bode plot of the step response of the MRC.

To quantify the system’s dynamic response capability, a frequency domain analysis was performed on the step response of the MRC under the model reference PID feedforward control. The resulting Bode plot is shown in Figure 8. This plot characterizes the equivalent frequency response of the system under open-loop feedforward control. Taking the −3 dB point as the control bandwidth benchmark, the Bode plot indicates a bandwidth of approximately 20 rad/s (≈3.2 Hz) for the MRA. This bandwidth is adequate for interactive tasks demanding high safety and compliance, such as slow physical human–robot interaction, compliant grasping, and posture maintenance. However, this bandwidth also highlights a fundamental limitation of the current purely feedforward control strategy. The dynamic performance is ultimately constrained by the intrinsic field establishment time of the MR material and eddy current effects in the magnetic circuit. The lack of real-time torque feedback prevents the system from effectively suppressing higher-frequency dynamic disturbances. This finding provides a crucial explanation for the performance degradation observed in the following section during high-frequency dynamic torque tracking experiments.

### 4.3. Dynamic Torque Output Experiment

**Figure 9:**
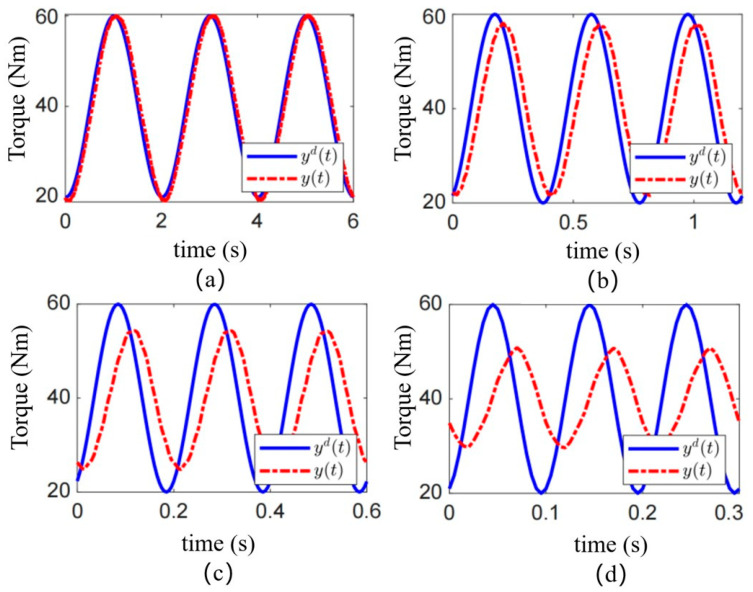
Control performance of sinusoidal desired torques at different frequencies. Sinusoidal torque at (**a**) 0.5 Hz; (**b**) 2.5 Hz; (**c**) 5.0 Hz; and (**d**) 10.0 Hz.

To evaluate the torque tracking performance of the MRA under dynamic operating conditions, sinusoidal desired torque commands at frequencies of 0.5 Hz, 2.5 Hz, 5.0 Hz, and 10 Hz were applied. The resulting clutch torque, obtained under the model reference PID feedforward control based on the fractional-order multi-state differential model, is shown in Figure 9. The solid blue line represents the desired torque. The pink/magenta dashed line represents the measured clutch torque. These results systematically reveal the performance boundaries of the proposed control strategy and their underlying causes.

Effective Low-Frequency Tracking and Model Validity: Under the low-frequency condition of 0.5 Hz, as shown in Figure 9a, the output torque of the MRC demonstrates excellent tracking of the desired sinusoidal waveform. No significant amplitude attenuation or phase lag is observable. This confirms that at relatively slow dynamics, the equivalent magnetic circuit model and the Bingham model established in Section 2 effectively characterize the system’s input-current-to-output-torque relationship. In this regime, the model-based feedforward control alone is sufficient to provide accurate commands, allowing the actuator to exhibit good dynamic compliance.

Performance Degradation at Medium and High Frequencies: As the frequency increases, the control performance degrades markedly. In Figure 9b, a slight phase lag and minor amplitude attenuation become apparent between the actual and desired torque at 2.5 Hz. This degradation becomes pronounced at 5.0 Hz (Figure 9c) and severe at 10 Hz (Figure 9d). Under the 10 Hz condition, the output torque exhibits substantial phase lag and its amplitude is significantly lower than the commanded value. The actuator exhibited high-fidelity tracking under a 0.5 Hz sinusoidal command, with an RMSE as low as 0.45 Nm. Effective tracking was maintained at 2.5 Hz (RMSE of 1.8 Nm).

This frequency-dependent performance deterioration fundamentally stems from the current model’s inability to fully capture the rate-dependent hysteresis nonlinearities of the MR fluid under rapidly changing magnetic fields. The primary contributing factors are: (1) Eddy Current Effects: Under high-frequency current excitation, eddy currents are induced within the ferromagnetic materials of the magnetic circuit. These currents generate an opposing magnetic field that delays the establishment of the intended magnetic field. Consequently, the actual magnetic flux density in the MR fluid lags behind the input current variation, directly causing the observed phase lag in the torque output. (2) Dynamic Yield Process of the MR Fluid: The phase transition of the MR fluid from a liquid to a semi-solid state itself requires finite time. In a rapidly alternating shear field, the formation and breakdown of chain-like structures involve dynamic response processes, introducing frequency-dependent energy dissipation and phase delay. These dynamics are not captured by a purely static model like the Bingham model. (3) Inherent Limitation of Open-Loop Control: The model reference PID is an open-loop feedforward strategy. Its performance is entirely dependent on the accuracy of the model. In the presence of the unmodeled dynamics mentioned above (eddy currents, dynamic yield), the controller cannot perceive the resulting torque tracking errors nor perform real-time compensation. Therefore, model inaccuracies are amplified under high-frequency dynamic conditions, directly leading to the severe performance collapse observed in Figure 9c,d.

### 4.4. Discussion

The experimental results presented in Section 4 directly validate and reflect the modeling and design decisions made in Section 2 and Section 3. The excellent steady-state torque tracking performance (Figure 7) and the high maximum output torque (153.7 Nm, close to the predicted value in Table 2) fundamentally validate the accuracy of the equivalent magnetic circuit model and the effectiveness of the genetic algorithm optimization. The optimization successfully balanced the magnetic saturation limits and thermal constraints, resulting in a prototype that delivers high torque without overheating under continuous operation within its specified range. Conversely, the degradation in dynamic tracking performance at higher frequencies (Figure 9) reveals the limitations of our current dynamic model and control strategy for high-speed scenarios. The dynamic model in Section 3.1, while capturing the essential rigid-body dynamics, does not fully incorporate the complex, rate-dependent hysteresis nonlinearities of the MR fluid. These unmodeled dynamics are the primary reason why the simple model reference PID feedforward controller struggles at frequencies above 2.5 Hz, leading to significant phase lag and amplitude attenuation.

There is a performance ceiling imposed by the open-loop, model-based control under high-frequency dynamics. Future work will therefore focus on (1) integrating more sophisticated hysteresis models (e.g., Bouc–Wen, Preisach) into the dynamic model and (2) developing hybrid control strategies that combine the robustness of model-based feedforward with a disturbance observer or weak feedback signals to enhance dynamic tracking performance without requiring a full torque sensor. While the static model-based reference feedforward control is highly effective for steady-state and low-frequency dynamic scenarios, its performance bandwidth is limited by the inherent complex dynamics of the magneto-rheological system. To enhance the high-frequency torque tracking performance of the MRA, future work must focus on integrating more advanced dynamic models capable of describing rate-dependent hysteresis (e.g., Bouc-Wen model, Preisach model) into the system model and may require introducing weak feedback strategies based on disturbance observers to compensate for model uncertainties.

## 5. Conclusions

This study presented an integrated solution for the design and control of a Magneto-Rheological Actuator, addressing the combined need for high torque, high compliance, and safety in flexible robotic joints. Through multi-physics modeling of the core MRC component using equivalent magnetic and thermal circuit models and employing a genetic algorithm for parameter optimization under multiple constraints including mechanical structure, magnetic saturation, maximum current, and maximum temperature, a prototype was successfully developed. The optimized MRA achieved a maximum output torque of 153.7 Nm, validating the effectiveness of the design approach. For application scenarios lacking torque sensors, a model-reference PID feedforward control strategy was designed. The experimental results show that it has good torque tracking performance. In static step response, the steady-state tracking error is tightly bounded within ±0.5 Nm (<0.33% of the full-scale range). The RMSE was calculated to be 0.21 Nm. Regarding dynamic tracking performance, the actuator exhibited high-fidelity tracking under a 0.5 Hz sinusoidal command, with an RMSE as low as 0.45 Nm. Effective tracking was maintained at 2.5 Hz (RMSE of 1.8 Nm). However, performance degraded at higher frequencies due to unmodeled rate-dependent hysteresis dynamics, clearly defining the bandwidth limitation (approximately 3.2 Hz) of the current model-based feedforward control. This research shows a complete chain from multi-physics modeling and parameter optimization to sensorless torque control, providing a practical and integrated solution for flexible robotic joints. Future work will focus on two main avenues: first, integrating more advanced dynamic hysteresis models into the control strategy to enhance mid-to-high-frequency dynamic performance; second, applying the designed MRA to quadruped robot or humanoid robot to validate its comprehensive performance in real-world tasks and promote its practical applications.

## Figures and Tables

**Table 1:** Nomenclature for the magnetic circuit model.

Symbol	Description
$T_{c}$	Engagement torque of magneto-rheological clutch
τ(·)	Shear stress of magneto-rheological fluid under different magnetic field strengths
B(x)	Nonlinear magnetic induction intensity in magneto-rheological fluid
N	Number of magneto-rheological fluid gaps
$N_{r}$	Number of rotor disks
$N_{s}$	Number of stator disks
$r_{i}$	Inner diameter of annular stator disks
$r_{o}$	Outer diameter of annular rotor disks
$\tau_{0}(\cdot )$	Yield stress of magneto-rheological fluid under different magnetic field strengths
η(·)	Viscosity coefficient of magneto-rheological fluid under different magnetic field strengths
$\dot{\gamma }(x)$	Shear rate of magneto-rheological fluid at radius x
h	Thickness of magneto-rheological fluid gap
${\dot{\theta }}_{m}$	Motor speed of magneto-rheological fluid joint
${\dot{\theta }}_{L}$	Rotational speed of load-connecting rod
G	Reduction ratio of reducer
$T_{B}$	Magneto-rheological torque
$T_{\eta }$	Viscous torque

**Table 2:** Validation of the optimization.

T	$t_{m}$	$t_{0}$	$B_{m}$	$B_{c}$	$B_{rc}$
153.7 Nm	124.7 °C	87.5 °C	1.005 T	1.8115 T	1.5676 T

## Data Availability

The original contributions presented in this study are included in the article. Further inquiries can be directed to the corresponding author.
